# Low-Coverage Whole Genomes Reveal the Higher Phylogeny of Green Lacewings

**DOI:** 10.3390/insects12100857

**Published:** 2021-09-23

**Authors:** Yuyu Wang, Ruyue Zhang, Yunlong Ma, Jing Li, Fan Fan, Xingyue Liu, Ding Yang

**Affiliations:** 1 College of Plant Protection, Hebei Agricultural University, Baoding 071001, China; zry47534@163.com (R.Z.); lijing1976@hebau.edu.cn (J.L.); auhff@hotmail.com (F.F.); 2 Department of Entomology, China Agricultural University, Beijing 100193, China; bjfuconidium@126.com; 3 College of Resources and Environmental Engineering, Mianyang Normal College, Mianyang 621000, China

**Keywords:** low-coverage genome, Chrysopidae, phylogenetic relationship, divergence time

## Abstract

**Simple Summary:**

Green lacewings (Chrysopidae) are one of the most commonly observed natural predators belonging to Neuroptera. They are widely distributed all over the world. The relationships among the three subfamilies of Chrysopidae have been controversial for a long time. We newly sequenced and analyzed the low-coverage genomes of five species (*Apochrysa matsumurae*, *Chrysopa pallens*, *Chrysoperla furcifera*, *Italochrysa pardalina*, *Nothochrysa sinica*), representing all three subfamilies, in order to reconstruct the higher phylogeny within this family. Our results suggested that Chrysopinae are a monophyletic sister group to the branch Apochrysinae + Nothochrysinae, and that Chrysopinae diverged from Apochrysinae + Nothochrysinae during the Early Cretaceous period (144–151 Ma), while Aporchrysinae diverged from Nothochrysinae around 117–133 Ma.

**Abstract:**

Green lacewings are one of the largest families within Neuroptera and are widely distributed all over the world. Many species within this group are important natural predators that are widely used for the biological control of pests in agricultural ecosystems. Several proposed phylogenetic relationships among the three subfamilies of Chrysopidae have been extensively debated. To further understand the higher phylogeny as well as the evolutionary history of Chrysopidae, we newly sequenced and analyzed the low-coverage genomes of 5 species (*Apochrysa matsumurae*, *Chrysopa pallens*, *Chrysoperla furcifera*, *Italochrysa pardalina*, *Nothochrysa sinica*), representing 3 subfamilies of Chrysopidae. There are 2213 orthologs selected to reconstruct the phylogenetic tree. Phylogenetic reconstruction was performed using both concatenation and coalescent-based approaches, based on different data matrices. All the results suggested that Chrysopinae were a monophyletic sister group to the branch Apochrysinae + Nothochrysinae. These results were completely supported, except by the concatenation analyses of the nt data matrix, which suggested that Apochrysinae were a sister group to Chrysopinae + Nothchrysinae. The different topology from the nt data matrix may have been caused by the limited sampling of Chrysopidae. The divergence time showed that Chrysopinae diverged from Apochrysinae + Nothochrysinae during the Early Cretaceous period (144–151 Ma), while Aporchrysinae diverged from Nothochrysinae around 117–133 Ma. These results will improve our understanding of the higher phylogeny of Chrysopidae and lay a foundation for the utilization of natural predators.

## 1. Introduction

Green lacewings (Neuroptera: Chrysopidae) are one of the most species-rich families within Neuroptera, comprising at least 1416 species from 82 genera, with new species still being discovered [1,2]. They are widely distributed all over the world, except Antarctica [3]. The larvae of Chrysopidae prey on field pests, such as aphids, psyllids and scale insects, which are widely used for biological control in different ecosystems (such as *Chrysoperla* Steinmann, 1964 and *Mallada* Navás, 1925) [4,5]. Most adult green lacewings do not feed on insects, except the species from the genera *Charysopa* Leach, 1815, and *Plesiochrysa* Adams, 1982, which are believed to be carnivorous [3,6].

The campodeoid-form larvae of some Chrysopidae species decorate themselves by carrying a packet of debris, which allows them to prey more easily and protect them from predators and parasites [7,8]. This debris-carrying behavior was an ancient characteristic found extensively in Mesozoic Cretaceous amber [9,10,11]. Adults are usually green, with metallic-luster compound eyes and transparent wings full of veins. The veins of green lacewings are typical characteristics, distinguishing them from other groups of Neuroptera [3].

Chrysopidae are traditionally divided into three extant subfamilies (Apochrysinae, Nothochrysinae, and Chrysopinae) and an extinct subfamily (Limaiinae), according to the characteristics of their veins, head, and genitalia [12]. Chrysopinae are the biggest subfamily, containing about 1352 species from 70 genera, occupying approximately 97% of Chrysopidae [13]. They are divided into four tribes: Belonopterygini, Leucochrysini, Ankylopterygini and Chrysopini, of which Chrysopini are the most species-rich, followed by Belonpterygini (15 genera), Leucochrysini (7 genera), and Ankylopterygini (6 genera) [14,15]. Nothochrysinae contain nine extant genera with some plesiomorphic characteristics, such as the presence of a forewing jugal lobe [3,16,17,18,19]. Although there are few living species in this subfamily, their fossil records from Late Cretaceous and Cenozoic periods are abundant compared to other subfamilies [16,20,21]. The decline of diversity in this subfamily was imputed to bat predation and global climate change [21]. Apochrysinae follow a typical pan-tropical distribution; containing 26 species of 5 genera, which are characterized by the absence of a forewing 1sc-r crossvein and an intra-median cell (im) [3,22,23].

The phylogenetic relationships within Chrysopidae have been debated for a long time. There are three hypotheses about the relationships among the three living subfamilies of Chrysopidae. Nothochrysinae were considered as a sister group to the rest of Chrysopidae because they exhibit many plesiomorphic characteristics, such as the absence of a tympanum at the base of the wing, the presence of a jugal lobe, and relatively unmodified wing venation [16,24,25]. This topology was also supported by Winterton et al. (2019) based on anchored hybrid enrichment data [26]. On the other hand, the proposition that Apochrysinae are a sister group to the rest of Chrysopidae was supported by the observation of some morphological characteristics as well as the mitochondrial genome [3,16,27]. Winterton and de Freitas (2006) also supported this hypothesis based on two mitochondrial genes (*COI*, *16S rDNA*) and one nuclear gene (*CAD*) [28]. More recent studies based on nuclear genes have suggested another hypothesis that Apochrysinae and Nothochrysinae formed a single branch, which is a sister group to Chrysopinae [29,30]. Jiang et al. (2017) also supported this hypothesis based on the complete mitochondrial genome [31]. Interestingly, Garzón-Orduña et al. (2019) obtained different topologies using different methods based on the molecular supermatrix. Their BI results suggested that Apochrysinae are a sister group to Chrysopinae + Nothochrysinae, whereas their ML results suggested that Chrysopinae are a sister group to Apochrysinae + Nothochrysinae [13]. So far, there is no consistent conclusion been drawn as to the phylogenetic relationships among the three subfamilies.

In recent years, next generation sequencing (NGS) has greatly improved the collection of orthologs for phylogenomic studies [32,33,34,35]. Novel methods of phylogenomic inference (e.g., coalescent-based inference [36,37,38]), evolutionary models (e.g., the site-heterogeneous model [39,40]), as well as new parameters for phylogenetic tree evaluation (e.g., internode certainty (IC) and related measures [41,42]), have also greatly improved our ability to reconstruct phylogenetic trees from genomic data.

In this study, we combined the power of low-coverage whole genome data with recently developed methods of phylogenetic inference to reconstruct and evaluate the higher-level phylogeny of Chrysopidae. We newly sequenced and analyzed the low-coverage genome of 5 species (*Apochrysa matsumurae*, *Chrysopa pallens*, *Chrysoperla furcifera*, *Italochrysa pardalina*, *Nothochrysa sinica*), representing 3 subfamilies of Chrysopidae, and used *Propylea japnonica* (Thunberg, 1781) (Coleoptera: Coccinellidae) as an outgroup to reconstruct the phylogeny of this family based on 2213 orthologs. The divergence time of the major lineages was estimated based on the topology recovered. The results will improve our understanding of the higher phylogeny of Chrysopidae and lay a foundation for the utilization of natural predators.

## 2. Materials and Methods

### 2.1. Insect Samples, DNA Extraction, and Sequencing

The collection information of all the specimens used in this experiment are summarized in Table 1. The species were identified by Yunlong Ma, based on morphological and genital characteristics. All the specimens were preserved in absolute ethanol at −20 °C. The total DNA was extracted from a single individual from each species using QIAamp DNA Micro Kits (QIAGEN, Stockach, Germany). DNA contamination and degradation was monitored with 1% agarose gels. Other quality parameters, such as purity, concentration, and integrity, were examined using a NanoPhotometer^®^ spectrophotometer (IMPLEN, Los Angeles, CA, USA) and a Qubit^®^ RNA Assay Kit in a Qubit^®^2.0 Fluorometer (Life Technologies, Carlsbad, CA, USA). Paired-end libraries were constructed with an insert size of 300 (2 × 150) bp, sequenced by a Illumina NovaSeq 6000 system from Majorbio (Shanghai, China). Approximately 40 G of raw data were produced for each library.

### 2.2. Genome Assembly

The rapid genome assemblies were conducted using the pipeline PLWS (http://github.com/xtmtd/PLWS, accessed on 30 October 2019), following Zhang et al. (2019) [35]. Firstly, quality control and normalization were conducted using BBTools v37.93 [43], and error correction was conducted using a Lighter v1.1.1 [44]. Next, contig assembly was performed using Minia v3.00-alpha1 [45], while redundancy removal was performed using Redundans v0.13c [46]. Finally, scaffolding was performed using BESST v2.2.8 [47] and gap filling was performed using GapCloser v1.12 [48]. The genome size was estimated using GenomeScope v1.0.0 [49].

### 2.3. Gene Alignment and Data Matrix Construction

*P. japonica* (Coleoptera: Coccinellidae) was selected as the outgroup. The ingroup taxa included five species of Chrysopidae (Neuroptera), which represented three subfamilies within this family (Table 2). The genome data of *P. japonica* was downloaded from the GenBank genome database (http://www.ncbi.nlm.nih.gov, accessed on 20 December 2019) with the accession number GCA_013421045.1 [50]. We used BUSCO v3.0.2 with endopterygota_odb9 (*n* = 2442) [51,52] to retrieve single-copy orthologs. The nucleotide (nt) sequences of all the orthologs were translated to amino acid (aa) sequences. All the orthologs were aligned by MAFFT v7.182 [53], based on their aa sequence, using L-INS-i. Next, PAL2NAL [54] was used to translate the aa sequence alignments to codon sequence alignments, and trimAl [55] was used with “automated1” to trim the aa sequence alignments. The trimmed segments of the aa sequence alignments were deleted from their corresponding codon sequence alignments using custom Perl scripts. We used BaCoCa [56] to detect the compositional heterogeneity and bias (RCFV value), and then aa with RCFV values smaller than 0.1 were selected to reconstruct the phylogenetic tree.

### 2.4. Heterogeneous Sequence Divergence Test

We used AliGROOVE [57] to test the extent of sequence similarity and alignment ambiguity in pair-wise sequence comparisons derived from the nt and aa data matrix. AliGROOVE establishes pair-wise comparisons of sequence divergences for each terminal against all other sequences in a multiple sequences alignment. The resulting distances matrix is then compared to the similarity of the whole alignment. The scores range from −1 (full random similarity) to +1 (nonrandom similarity). All analyses were conducted under the default parameters.

### 2.5. Phylogenetic Inference

For the codon sequence and aa alignments of each gene, the un-rooted phylogenetic tree under the optimality criterion of maximum likelihood (ML) was inferred using IQ-TREE v1.6.10 [58] with automatically selected best models. For the concatenation analysis, the codon sequence and aa alignments from all genes were analyzed as a single super-matrix. The concatenated file was partitioned based on every gene, and the model for every gene was the automatically selected best model. Node support values were calculated using 1000 SH-aLRT replicates and 1000 ultrafast bootstraps [59,60]. Individual gene trees for each gene alignment were estimated with IQ-TREE with automatically selected best models and analyzed with ASTRAL-III v5.6.1 [61] to infer the coalescent-based species trees, with local branch supports estimated from quartet frequencies [62]. In order to reduce the influences of the heterogeneity of the data matrix, we reconstructed the phylogenetic tree using the aa data matrix under the heterogeneous model (LG + C60 + F), as well as selecting the aa with RCFV values smaller than 0.1. The trees were visualized and edited using FigTree v1.3.1 (https://github.com/rambaut/figtree/releases, accessed on 18 December 2020).

### 2.6. Likelihood Mapping Analysis

The phylogenetic information on aa data matrix, as well as the data matrix of aa with RCFV values smaller than 0.1, were implemented using the likelihood mapping approach [63]. Four clusters were specified according to the phylogenetic tree obtained: cluster 1 represented Chrysopinae, including *C. pallens*, *Ch. furcifera* and *I. pardalina*; cluster 2 represented Apochrysinae, including *A. matsumurae*; cluster 3 represented Nothochrysinae, including *N. sinica*; and cluster 4 represented the outgroup, including *P. japonica*. The number of quartets to be randomly drawn was set to 2000, and the subsequent tree search was skipped.

### 2.7. Divergence Time Estimation

Divergence times were calculated using mcmctree in PAML [64]. We used the aa with RCFV values smaller than 0.1 and the topology obtained with all analyses except the nt data marix from the phylogenetic analysis. Single orthologous genes were concatenated into 37 megagene, according to the best-fit model determined by ModelFinder [65]. Two independent runs were conducted using the independent rates model, with 200,000 generations kept, 50,000 generations discarded as burn-in, and sampfreq 10. Two fossil records were used to calibrate the node ages of the branch Coloptera + Neuroptera (298.9–279.3 million years ago) as well as the branch of Chrysopidae (157.3–145.0 million years ago), according to the PBDB database (https://paleobiodb.org/navigator/, accessed on 24 December 2020).

## 3. Results

### 3.1. Genome Assembly

**Figure 1:**
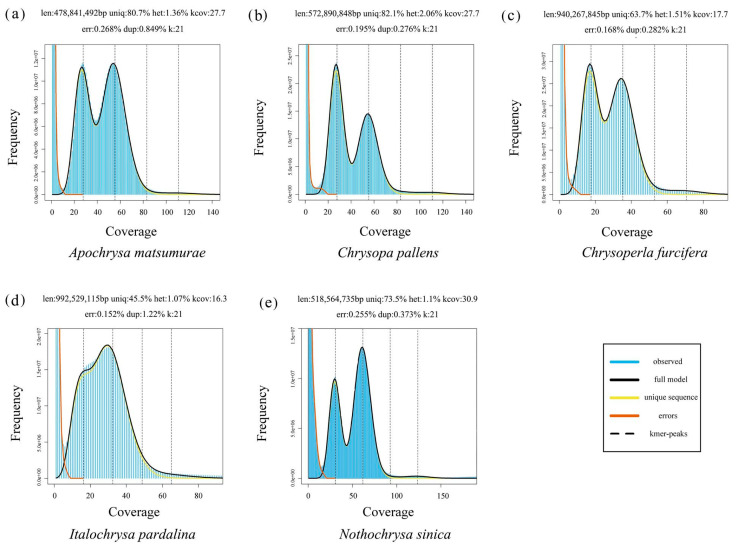
Characteristics of newly sequenced genomes in this study inferred using GenomeScope. len: genome haploid length; uniq: percentage of the genome unique length; het: heterozygosity; kcov: coverage of hybrid peak; err: read error rate; dup: duplicates; k: k-mer size.

The raw data for every species was 40 G. The number of paired-end tags for each of the five species was 148,875,292 for *A. matsumurae*, 164,308,429 for *C. pallens*, 165,447,806 for *I. pardalina*, 164,625,294 for *Ch. furcifera*, and 165,186,723 for *N. sinica*, respectively, after cleaning. The average read coverage of every species was more than 40X. The estimated genome sizes of the newly sequenced species were about 479 Mb for *A. matsumurae*, 573 Mb for *C. pallens*, 940 Mb for *Ch. furcifera*, 992 Mb for *I. pardalina*, and 519 Mb for *N. sinica*, respectively (Table 2, Figure 1). The heterozygosity ranged from 1.07% for *I. pardalina* to 2.06% for *C. pallens*. The genomes’ repeat lengths ranged from 92.31 Mb for *A. matsumurae* to 540.20 Mb for *I. pardalina*. The genomes’ unique lengths ranged from 380.87 Mb for *N. sinica* to 598.49 Mb for *Ch. furcifera*.

**Figure 2:**
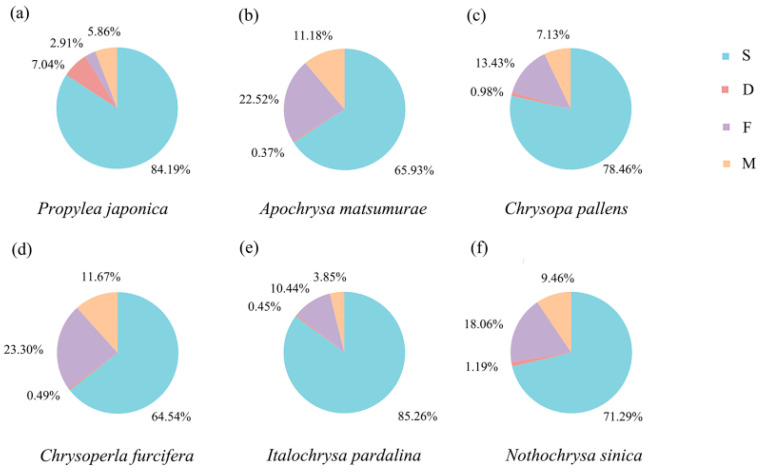
Summarized benchmarking of BUSCO notations for species used in this study. S: complete and single-copy BUSCOs; D: complete and duplicated BUSCOs; F: fragmented BUSCOs; M: missing BUSCOs. The different colors represent the different kind of BUSCOs, accordingly. The numbers indicate the percentage of different kinds of BUSCOs within the total number BUSCO groups searched.

The five species of Chrysopidae with newly sequenced low-coverage genomes were used as the ingroups and *P. japonica* was selected as the outgroup to construct the phylogenetic data matrix. Single-copy orthologs were retrieved using the BUSCO v3.0.3 with endopterygota_odb9 (*n* = 2442), as previously described [51]. The number of complete and single-copy orthologs for every species were 2056 for *P. japonica*, 1610 for *A. matsumurae*, 1916 for *C. pallens*, 1576 for *Ch. furcifera*, 2082 for *I. pardalina*, and 1741 for *N. sinica*, accounting for 84.19, 65.93%, 78.46%, 64.54%, 85.26%, and 71.29% of the total BUSCO groups searched, respectively (Table 3 and Figure 2). In total, 2213 orthologs present in more than 3 species were selected to construct the phylogenomic data matrix. The nt concatenated data matrix contained 3,126,564 sites, and the aa concatenated data matrix contained 1,042,188 sites. Finally, 439 orthologs passed the BaCoCa test with a relative composition frequency variability (RCFV) value smaller than 0.1 [56].

### 3.2. Heterogeneous Sequence Divergence

**Figure 3:**
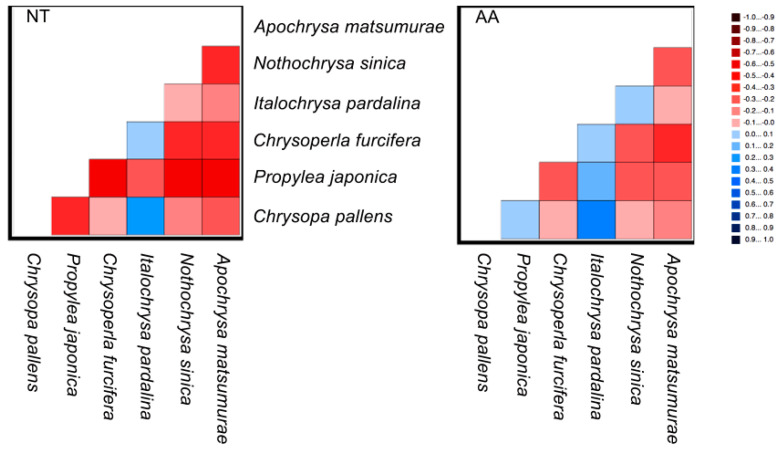
AliGROOVE analyses of nucleotide (NT) and amino acid (AA) sequences. The mean similarity score between the sequences is represented by a colored square, based on AliGROOVE scores from −1, indicating a great difference in rates from the remainder of the dataset (i.e., heterogeneity (red), to +1, indicating rates matching those from all the other comparisons (blue).

The results from the AliGROOVE analyses demonstrated strong heterogeneity in pair-wise sequence comparisons derived from the nt and aa data matrices (Figure 3). In particular, the pair-wise sequence comparisons of nt data yielded extremely low scores in almost all species, while pair-wise sequence comparisons of aa data received relatively higher scores.

### 3.3. Phylogenetic Analysis

**Figure 4:**
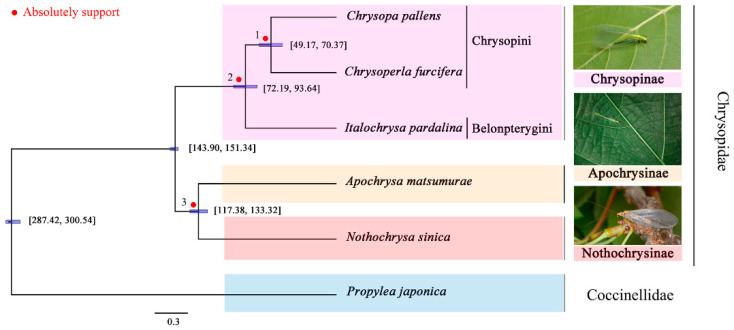
Phylogenetic reconstructions and divergence times of Chrysopidae, based on amino acids with relative composition frequency variability (RCFV) values smaller than 0.1. The concatenated and coalescent analyses of aa as well as the coalescent analyses of nucleotides (NT) all supported this topology. The red dot indicates complete support for the node in all the analyses mentioned above (Node 1, 2, 3). The numbers near each node are the 95% HPD values.

Both concatenation and species coalescence analyses of the aa data matrix, as well as the species coalescence analyses of the nt data matrix under the best-fit model determined by ModelFinder, presented the same topology (Table 4, Figure 4) [65]. Within Chrysopidae, Apochrysinae were found to be the sister group to Nothochrysinae, with complete support from the data. Chrysopinae were found to be a monophyletic sister group to the branch Apochrysinae + Nothochrysinae, with complete support. When using both the heterogeneous model (LG + C60 + F) and aa with RCFV values smaller than 0.1, the aa presented the same topology as in the concatenation and species coalescence analyses of the aa data matrix. However, the concatenation analyses of the nt data matrix showed Chrysopinae to be a sister group to Nothchrysinae, and Apochrysinae as be a sister group to the branch Chrysopinae + Nothchrysinae (Figure S1).

### 3.4. Likelihood Mapping Analysis

**Figure 5:**
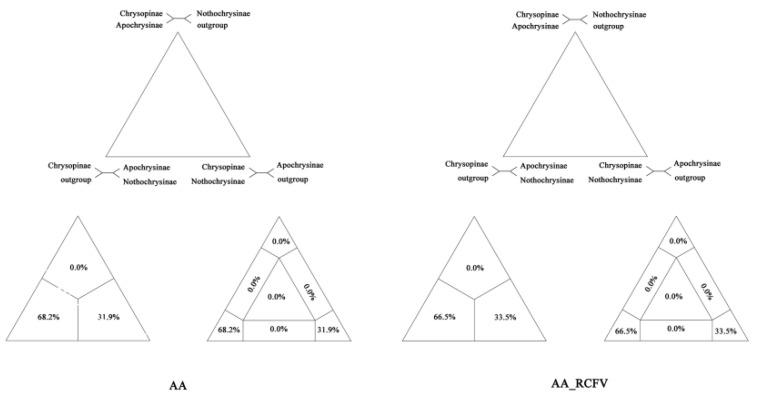
Likelihood mapping results for the amino acids (aa) data matrix, as well as the aa with relative composition frequency variability (RCFV) values smaller than 0.1. AA: amino acids; AA_RCFV: aa with relative composition frequency variability values smaller than 0.1.

The likelihood mapping analysis suggested both a sister group relationship between Nothchrysinae and Apochrysinae, and the formation of a sister group to Chrysopinae by Nothchrysinae and Apochrysinae together (68.2% for the aa data matrix and 66.5% for aa with RCFV values smaller than 0.1) (Figure 5). Alternative relationships, such as Apochrysinae being a sister group to Chrysopinae + Nothochrysinae, were weakly supported (31.9% for aa data matrix and 33.5% for aa with RCFV values smaller than 0.1). The results were consistent with our phylogenetic analysis, and the data matrix used here were absolutely informative. Thus, we used this topology, obtained using aa with RCFV values smaller than 0.1, for the estimation of the divergence time.

### 3.5. Divergence Time Estimation

The percentage of the total deviation for all branches in the two runs was smaller than 0.1%, ensuring they converged (Figure 4 and Figure S2). The chronogram in Figure 4 presents the divergence time estimation (as median node heights), based on the topology recovered from the phylogenetic tree. The 95% highest posterior density (HPD) values of each node were calculated. Our phylogenomic study suggested that Neuroptera diverged from Coleoptera during the Early Permian period (287–301 Ma). Chrysopinae diverged from Apochrysinae + Nothochrysinae during the Early Cretaceous period, (144–151 Ma) while Aporchrysinae diverged from Nothochrysinae around 117–133 Ma. Belonpterygini diverged from Chrysopini during the Late Cretaceous period (72–94 Ma).

## 4. Discussion and Conclusions

The dramatically decreased cost of low-coverage whole genome sequencing has facilitated the generation of genome-scale data from a wide variety of organisms. To date, there are more than 600 insect genomes sequenced and available at GenBank (https://www.ncbi.nlm.nih.gov/genome/browse#!/overview/insects, accessed on 24 June 2021). These large datasets undoubtedly provide significant molecular evidence for understanding the phylogeny and evolution of insects. In this study, five low-coverage genomes of Chrysopidae were sequenced and analyzed. There were no specific size patterns or genome characteristics at the level of subfamilies.

All the data matrices suggestd that Chrysopinae are a monophyletic sister group to Apochrysinae + Nothochrysinae, except the concatenation analyses of the nt data matrix, which suggested that Apochrysinae are a sister group to Chrysopinae + Nothchrysinae. The different topology from the nt data matrix may have been caused by the limited sampling of Chrysopidae. Our results supported the conclusions from previous quantitative analyses based on molecular data [29,30,31]. Nothochrysinae were believed to be a sister group to the rest of Chrysopidae for a long time because they shared numerous supposed plesiomorphic characteristics and left more fossil records than both Apochrysinae and Nothchrysinae [3,12,13,16,21,24,25]. However, there are no published studies based on molecular data (including our study here) supporting this hypothesis up to now. Chrysopinae was demonstrated diverged from Apochrysinae + Nothochrysinae during the Early Cretaceous period (144–151 Ma), while Aporchrysinae diverged from Nothochrysinae around 117–133 Ma. The aa data were more effective than nt and coalescence analyses were more suitable in dealing with the limited sampling in the phylogenetic analyses of our study. More comprehensive samplings are needed to explore the higher phylogeny of Chrysopidae in the future.

## Figures and Tables

**Table 1:** Collection information on species used in this study.

Order/Family/Subfamily	Species	Voucher Code	Date	Place	Collection Method	Collector
Neuroptera						
Chrysopidae						
Apochrysinae	*Apochrysa matsumurae*	AYX001	2010-VIII-8	Hachioji Minamiosawa TMU Campus, Tokyo, Japan	collected by net	Xingyue Liu
Chrysopinae	*Chrysopa pallens*	WYY1	2019-I-23	Reared at Langfang experiment base of Chinese Academy of Agricultural Sciences, Langfang, China		Mengqing Wang
	*Chrysoperla furcifera*	CHR003	2018-IX-6	Shibatan, Mt Wuling, Hebei, China	collected by net	Xingyue Liu
	*Italochrysa pardalina*	CHR002	2013-V-15	Academy of Forestry, Nanning, Guangxi, China	collected by net	Xingyue Liu
Nothochrysinae	*Nothochrysa sinica*	CHR001	2018-VIII-6	Fengxian Jialingjiangyuan, Baojishi, Shannxi, China	trapped by light	Yingnan He

**Table 2:** Genomescope results of species used in this study.

Species	Heterozygosity (%)	Genome Haploid Length (Mb)	Genome Repeat Length (Mb)	Genome Unique Length (Mb)
*Apochrysa matsumurae*	1.362–1.366	478.62–478.84	92.31–92.35	386.31–386.49
*Chrysopa pallens*	2.057–2.059	572.85–572.89	102.27–102.28	470.58–470.61
*Chrysoperla furcifera*	1.509–1.510	940.17–940.27	341.68–341.72	598.49–598.55
*Italochrysa pardalina*	1.068–1.071	991.85–992.53	540.20–540.59	451.65–451.96
*Nothochrysa sinica*	1.096–1.097	518.51–518.56	137.64–137.66	380.87–380.91

**Table 3:** Summary of BUSCO results of species used in this study.

Species Name	S	D	F	M	T
*Propylea japonica*	2056	172	71	143	2442
*Apochrysa matsumurae*	1610	9	550	273	2442
*Chrysopa pallens*	1916	24	328	174	2442
*Chrysoperla furcifera*	1576	12	569	285	2442
*Italochrysa pardalina*	2082	11	255	94	2442
*Nothochrysa sinica*	1741	29	441	231	2442

S: Complete and single-copy BUSCOs; D: Complete and duplicated BUSCOs; F: Fragmented BUSCOs; M: Missing BUSCOs; T: Total BUSCO groups searched.

**Table 4:** Node support values of the finial phylogenetic tree, calculated using SH-aLRT replicates and ultrafast bootstraps.

Node	AA Concatenation Analyses	AA Species Coalescence Analyses	AA with RCFV Values Smaller Than 0.1	NT Species Coalescence Analyses
1	100/100	1	100/100	1
2	100/100	1	100/100	1
3	100/100	1	100/100	1

NT: nucleotide. AA: amino acid.

## Data Availability

The raw data and the assemblies were deposited in the National Center for Biotechnology Information, with the BioProject access number PRJNA759936.

## Supplementary Materials

The following are available online at https://www.mdpi.com/article/10.3390/insects12100857/s1. Figure S1: Phylogenetic reconstructions of Chrysopidae, based on concatenated nucleotides (NT). The numbers near each node are the supporting values, calculated using 1000 SH-aLRT replicates and 1000 ultrafast bootstraps; Figure S2: Divergence times of Chrysopidae, based on amino acids with relative composition frequency variability (RCFV) values smaller than 0.1. The numbers near each node are the 95% HPD values.
