# Supplementary material for: Low-Coverage Whole Genomes Reveal the Higher Phylogeny of Green Lacewings
Source: Insects. 2021 Sep 23;12(10):857. doi: 10.3390/insects12100857 (PMC8539002; doi:10.3390/insects12100857)
Supplement: Supplementary file 1 [file insects-12-00857-s001.zip › insects-1368423-supplementary.pdf]

Supplementary Materials: Figure S1: Phylogenetic reconstructions of Chrysopidae, based on concatenated nucleotides (NT). The numbers near each node are the supporting values, calculated using 1000 SH-aLRT replicates and 1000 ultrafast bootstraps; Figure S2: Divergence times of Chrysopidae, based on amino acids with relative composition frequency variability (RCFV) values smaller than 0.1. The numbers near each node are the 95% HPD values.

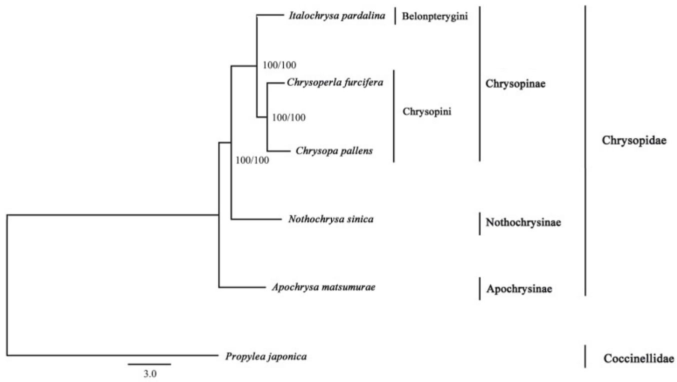

Figure S1. Figure S1: Phylogenetic reconstructions of Chrysopidae, based on concatenated nucleotides (NT). The numbers near each node are the supporting values, calculated using 1000 SH-aLRT replicates and 1000 ultrafast bootstraps

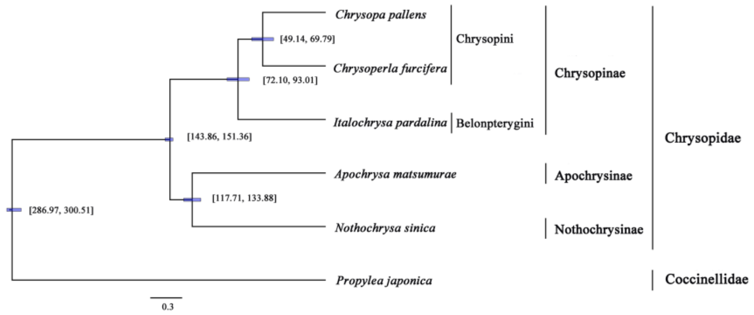

Figure S2. Divergence times of Chrysopidae, based on amino acids with relative composition frequency variability (RCFV) values smaller than 0.1. The numbers near each node are the 95% HPD values.
